# Enhancing clinical reasoning skills for medical students: a qualitative comparison of LLM-powered social robotic versus computer-based virtual patients within rheumatology

**DOI:** 10.1007/s00296-024-05731-0

**Published:** 2024-10-16

**Authors:** Alexander Borg, Benjamin Jobs, Viking Huss, Cidem Gentline, Fabricio Espinosa, Mini Ruiz, Samuel Edelbring, Carina Georg, Gabriel Skantze, Ioannis Parodis

**Affiliations:** 1 Division of Rheumatology, Department of Medicine Solna, Karolinska Institutet and Karolinska University Hospital, Stockholm, Sweden; 2 Division of Clinical Epidemiology, Department of Medicine Solna, Karolinska Institutet, Karolinska University Hospital Solna, D2:01 Rheumatology, Stockholm, SE-171 76 Sweden; 3 Department of Clinical Science, Intervention and Technology, Karolinska Institutet, Stockholm, Sweden; 4 School of Health Sciences, Örebro University, Örebro, Sweden; 5 Department of Neurobiology, Care Sciences and Society, Karolinska Institutet, Stockholm, Sweden; 6 Division of Speech Music and Hearing, Royal Institute of Technology, KTH, Stockholm, Sweden; 7 Department of Rheumatology, Faculty of Medicine and Health, Örebro University, Örebro, Sweden

**Keywords:** Patient simulation, Clinical reasoning, Rheumatology, Refractive index

## Abstract

Virtual patients (VPs) are increasingly used in medical education to train clinical reasoning (CR) skills. However, optimal VP design for enhancing interactivity and authenticity remains unclear. Novel interactive modalities, such as large language model (LLM)-enhanced social robotic VPs might increase interactivity and authenticity in CR skill practice. To evaluate medical students’ perceptions of CR training using an LLM-enhanced social robotic VP platform compared with a conventional computer-based VP platform. A qualitative study involved 23 third-year medical students from Karolinska Institutet, who completed VP cases on an LLM-enhanced social robotic platform and a computer-based semi-linear platform. In-depth interviews assessed students’ self-perceived acquirement of CR skills using the two platforms. Thematic analysis was employed to identify themes and sub-themes. Three main themes were identified: authenticity, VP application, and strengths and limitations. Students found the social robotic platform more authentic and engaging. It enabled highly interactive communication and expressed emotions, collectively offering a realistic experience. It facilitated active learning, hypothesis generation, and adaptive thinking. Limitations included lack of physical examination options and, occasionally, mechanical dialogue. The LLM-enhanced social robotic VP platform offers a more authentic and interactive learning experience compared to the conventional computer-based platform. Despite some limitations, it shows promise in training CR skills, communication, and adaptive thinking. Social robotic VPs may prove useful and safe learning environments for exposing medical students to diverse, highly interactive patient simulations.

**Supplementary Information:**

The online version contains supplementary material available at 10.1007/s00296-024-05731-0.

## Key message

•An LLM-powered social robotic VP platform provides a more authentic and interactive learning experience compared to conventional computer-based VPs.
•Medical students undertaking clinical placements within rheumatology experienced that an LLM-enhanced social robotic platform can provide added value in training CR skills, particularly through realistic communication.
•Social robotic VPs may prove useful and safe learning environments for exposing medical students to diverse, highly interactive patient simulations.

**Supplementary Information:**

The online version contains supplementary material available at 10.1007/s00296-024-05731-0.

## Introduction

A multitude of various interactive modalities are currently being used as tools for practicing specific learning outcomes in health professions education (HPE) curricula [1]. Virtual patient (VP) simulations constitute an example of such modalities. They are computerised case scenarios that allow users to interact with a platform presenting a patient case scenario in an educational context and are being increasingly used and evaluated [2, 3]. Studies have extensively examined the educational value of VPs, including investigation of how VP design contributes to the development of clinical reasoning (CR) skills [4, 5]. CR skills are essential for healthcare professionals to ensure patient safety through accurate diagnostic procedures and clinical decisions [6, 7]. The chronic and complex nature of the majority of rheumatic diseases makes this medical discipline particularly suitable for training students in CR [8–10]. Disease manifestations and presentations are often heterogeneous, presenting challenging scenarios for the training of differential diagnostics [11]. Moreover, some rheumatic conditions are rare [12], making it difficult to ensure that all students encounter patients with representative diagnoses during clinical rotations.

VPs are widely accepted as educational tools for training CR skills in a safe environment and are used as a complement to real patient encounters [13]. However, implementation of VPs is oftentimes complicated as it requires adjustments to specific contexts and learning outcomes, and clear recommendations for which VP platform best fits which setting has yet to be fully explored [14]. Recent advancements in artificial intelligence (AI) have led to the development of large language models (LLMs), such as the chat-generative pre-trained transformer (ChatGPT) that was introduced in late 2022 [15]. LLMs are AI models trained on vast amounts of text data, enabling them to generate human-like responses to prompts. The potential application of LLMs are being rapidly explored in various fields, including medical pedagogy [16]. Integrating LLMs with VP simulations has been proposed as a way to enhance medical education by presenting a higher volume of engaging clinical scenarios [17]. Some limitations with the integration of LLMs in VP simulations, which primarily has been using chatbots, include availability aspects and potential restrictions in conversational flow and authenticity compared to real-life patient interactions [18, 19].

Social robots are designed for human-robot interaction and can be applied and used within different contexts [20]. These robots are equipped with features that enable them to communicate and engage with users in a natural and intuitive way. Integration of LLMs into social robots has been shown to result in authentic interactions, as these robots can convey appropriate emotions through subtle facial expressions and gestures [21]. This integration has not been used to present VPs within medical education but may be hypothesised to enhance the development of CR skills by providing more complex and authentic interactions compared to traditional VP platforms [22].

The aim of this study was to explore the potential added value of a social robotic VP platform, enriched with an LLM, compared with a conventional computer-based platform for medical students practicing CR skills during clinical placements within rheumatology.

## Methods

We conducted an interventional explorative qualitative study to investigate how medical students perceived training in CR using a social robotic VP platform enhanced with an LLM compared with a conventional computer-based VP platform. Data for this comparison were derived from qualitative in-depth interviews. We followed the consolidated criteria for reporting qualitative research (COREQ) as a research reporting guideline checklist for qualitative studies [23]. The report is found in the Supplementary Table S1.

### Participants

Third-year medical students from Karolinska Institutet (KI) were recruited in this study. The recruitment took place during the course “Clinical medicine 2: applied internal medicine and related disciplines”, specifically while students were partaking in clinical placements within rheumatology at the Karolinska University Hospital.

Participants in the in-depth interviews (*n* = 23) were recruited from a pool of students who undertook the aforementioned course during the spring term of 2024 (*n* = 117). Of 60 students who consented to participate in the study, 23 students were finally included, determined by the chronological order of the participant response. The number of interviews was deemed sufficient as it yielded saturation during the thematic analysis, as per the COREQ recommendations [23].

Participation in the study was voluntary, and all participants provided written informed consent prior to inclusion. The Swedish Ethical Review Authority reviewed and approved the study protocol prior to initiation (registration number: 2022-04437-01; date of approval: December 12th, 2022).

### VP case development and practice

We developed ten VP cases to complement real patient encounters during the rheumatology placements. The cases were designed in the English language to facilitate availability for exchange medical students. The cases were presented using two different platforms: an LLM-empowered social robot from Furhat robotics [20] and a computer-based platform called Virtual Interactive Case simulator (VIC) [24]. Each platform contained four unique cases, and one case that was identical between the two platforms. The development of cases for both platforms adhered to previously proposed principles for VP case development [5]. The cases covered various rheumatic conditions, including rheumatoid arthritis (RA), polymyalgia rheumatica (PMR), gout, systemic lupus erythematosus (SLE), granulomatosis with polyangiitis (GPA), dermatomyositis (DM), and spondyloarthritis (SpA).

Following previous recommendations for enhancing CR skills through VP practice in dyads [25], students were instructed to work on the VP cases in pairs or small groups of three. Students completed each VP case when they subjectively considered that they had obtained sufficient information for proposing a diagnosis and a management plan for the virtual patient. Immediately after completing each one of the VP cases, the students participated in a follow-up seminar in groups. These seminars assembled student groups ranging between six and eight students and were led by a consultant rheumatologist. During the seminars, the students discussed differential diagnoses and any questions that arose during the VP practice. Half of the students started with the computer-based platform while the other half started with the social robotic platform. By the end of the clinical placement, all students had completed all VP cases in both platforms and had attended the corresponding follow-up seminars.

### The social robotic platform

The social robot has an animated face and a neck that allows realistic head movements, which we hypothesised would enhance the realism during VP encounters [20]. The robot displays subtle facial expressions and emotions, such as gaze behaviour, to indicate the patient’s emotional state [21]. We used the social robot by Furhat robotics, which integrates the Furhat software development kit (FurhatSDK) [20], and combined it with the OpenAI gpt-3.5-turbo language model [26]. To reflect the patient’s emotional state during the dialogue, we prompted the LLM to generate appropriate facial expressions at specific anchor points during the conversations [27]. The LLM selected appropriate facial expressions for each anchor point from a predefined set that is available through the FurhatSDK. This selection was based on the context of the VP’s responses, which were given in different emotional states on a stochastic basis.

To generate authentic VP responses from a patient’s perspective, preventing assistant-like phrasings, we created a detailed patient description and included it within the prompt. The VP’s responses incorporated information from this description, instructions on how to generate the dialogue line, as well as the last 10 dialogue turns between the students and the VP.

An issue that can arise when using LLMs in turn-taking dialogue is response delays [27]. To avoid misinterpretation of when it is possible to talk to the VP, we created a turn-holding cue to signal whether the robot is perceiving input by the students or is about to respond [28]. This was achieved by using an LED light at the bottom of the robot, indicating the turn-holding status with pre-specified colours.

Prior to starting a VP case in the social robotic platform, students were provided with introductory information about the case as well as results from relevant laboratory tests along with their corresponding reference values.

### The computer-based platform

VIC is a semi-linear interactive learning platform, within which users can explore VP case information in any order, while the case presentation (first step) and conclusion (last step) remain constant [29]. Users investigate the case by clicking on pre-written questions which lead into answers about the patient’s medical history. They can also perform virtual clinical investigations, such as physical examinations, and review laboratory results and assessment findings, including imaging. Once students perceived that they had obtained sufficient information from the patient history, investigations, and test results, they could conclude the case by making a diagnosis and deciding on a management plan for the VP.

### Data collection

After completing all the VP cases in both platforms, including the corresponding follow-up seminars, the students who had consented to the study participated in individual, semi-structured interviews. Those interviews followed an interview guide pertaining to learning experiences, the platforms’ contribution to CR training, strengths and limitations of each platform, and suggestions for improvements. The interview guide was drafted by AB under the supervision of IP and revised by MR, SE, CG, and GS based on a previously developed interview guide, used in the pilot testing of the social robotic modality, also drafted by AB and IP and revised by MR, SE, CG, and GS [22]. The interview guide is provided in the online Supplementary Figure S1.

The interviews were conducted by the same investigator (AB) either at the Division of Rheumatology at the Karolinska University Hospital or remotely via video calls. AB was at the time a PhD student within medical pedagogy and received supervision in qualitative methodology from senior researchers with experience in the field (CG, SE, and IP). Each interview lasted between 40 and 60 min. The interviews were recorded, fully transcribed, and pseudonymised. Upon removal of any personal or sensitive information, interview data were stored on secure servers accessible only to involved researchers.

### Analysis

The data were processed systematically using established methodology for thematic analysis [30, 31]. The thematic analysis employed a hybrid approach within a phenomenological framework. Initially, a deductive method was employed through development of codes that were aligned with international recommendations for integrating CR skill training in health professions education [6]. This provided a structured starting point grounded in established theory. To balance this with the phenomenological nature of the study, inductive elements were incorporated throughout the analysis process. During analysis and initial theme development, researchers (AB and BJ) remained perceptive to new themes that emerged from the data to capture unique aspects of the participants’ experience that could not be derived from the existing recommendations or frameworks.

The authors started by repeatedly reviewing the interviews to familiarise with the content. They first analysed each verbatim transcribed interview individually and then compared the interviews to contextualise emerging themes. The coding process was iterative and collaborative, involving multiple rounds of analysis. The researchers independently coded the data in their verbatim transcribed format and met regularly to compare and discuss their interpretations. Through these discussions, they rectified the coding scheme, resolved any discrepancies, and gradually refined the emerging themes until reaching consensus on the final thematic structure. The codes were used to highlight sections of the interviews relating to CR training. The bulk of the analysis and theme development were conducted by AB and BJ, under the supervision of IP who guided the selection of themes.

This approach allowed findings to be applicable within the broader context of CR education research, while remaining true to participants’ lived experiences. Throughout the analysis, reflexivity was maintained regarding how the initial coding framework influenced data interpretation, actively seeking out experiences that contradicted from or expanded upon existing theories. The authors had diverse and complemental backgrounds and expertise in HPE and technological aspects, contributing unique perspectives to the interpretation of the results.

### Patient and public involvement statement

Patient research partners (Yvonne Enman, Karin Blomkvist Sporre) were involved in the design of the study and are committed to disseminating the results upon publication.

## Results

Fourteen of 23 students (61%) were male and nine (39%) were female. Seventeen (74%) responded that they had no previous experience with VPs. The students’ mean age was 23.5 (standard deviation: 6.0) years. Twelve of the students (52%) started with the social robotic platform, whereas eleven (48%) started with the computer-based VPs. Thematic analysis resulted in three themes: (i) authenticity, (ii) VP application, and (iii) strengths and limitations. Each theme was further divided into sub-themes, totalling seven across all themes. Table 1 details the themes along with their corresponding sub-themes and analytical codes.

**Table 1:** Identified themes and sub-themes from within the qualitative thematic analysis

Themes	Sub-themes	Analytical codes
Authenticity	Communication	Interactivity
		Sensory input
		Immersion
	Realism	Proximity to real scenario
		Authenticity
		Feeling like a physician
	Medical history and examination	Medical history
		Data collection
		Physical examination
		Information
VP application	Learning facilitators	Groups
		Combination of platforms
		User-friendly
		Difficulty
		Language
	Skill development	Medical knowledge
		Decision-making
		Learning through failure
		Question generation
		Empathy
		CR training
		Hypothesis synthesis
Strengths and limitations	Technical limitations	Lack of examination
		Pre-written questions
		Lack of interactivity
		Listening time
	VP potential	Representative patients
		Experience
		Efficiency
		Complement to medical rotations
		Safety
		Availability

### Authenticity

The theme “authenticity” was divided into three sub-themes, i.e., (i) communication, (ii) realism, and (iii) medical history and examination.

Communication played a crucial role in creating an authentic experience for the students. The interactivity, sensory input, and immersion provided by the VP platforms, particularly the social robotic platform, contributed to a more engaging and authentic learning experience. Students reported that face-to-face interactions, the ability of the robot to ask questions and express emotions, and the presence of a real voice enhanced the sense of authenticity in the communication process. Relevant quotes are illustrated in Table 2.

**Table 2:** Relevant quotes by theme and sub-themes from in-depth interviews

Study participant	Quote
**Authenticity**	
Communication	
Student #22 (female)	“Yes. I think that these robots give more immersion than the computer-based cases. Because it is a face-to-face interaction. These robots interact as a patient. They ask questions and wonder. This is not done in the computer-based platform.”
Student #16 (male)	“It was a direct contact with the robot. Having a real voice does a lot. It felt a lot like a real patient visit”
Student #22 (female)	“[…] there was a feeling that it was more real when the robot moved or had a facial expression. You get the feeling that it was more about the patient than just reading a text.”
Student #20 (male)	“I think that the robot case was more interactive and by working on the case, you would remember more about the case.”
Realism	
Student #19 (female)	“You can tell that it’s not a real patient or person, but I was surprised by how well this robot could express its feelings. You could tell it was irritated or frustrated, and that it wanted a good evaluation or confirmation that I was listening. I think it’s easier to feel something for the robot case than for the computer platform. […] Here [with the robot], you know it’s not a real patient either. But it’s a bit more realistic.”
Student #5 (male)	“[…] the robot was more personal. The computer was good, but it wasn’t as personal. You could get a lot of information and understand who you were dealing with [using the robot], like with a regular patient.”
Student #11 (male)	“I think that the robot cases gave a more authentic experience. […] you get to talk to it, and that makes it easier to get into the role of a physician.”
Student #20 (male)	“You get to train more on medical history taking with the robot cases, […] when we did the computer cases, it was similar to the administrative work that you [as a physician] do after you have met a patient.”
Medical history and examination	
Student #21 (female)	“[…] with the robot case, there was also a little more communication practice, knowing which specific questions that can be good to ask and in what way. […] it was especially important to remember what the patient had said, […] I didn’t get that [feeling] in the same way with the computer cases […]”
Student #23 (female)	“If I wanted blood tests, I could get them right away in the computer case.”
Student #7 (female)	“Downsides with the computer cases are that you see what questions you can choose to ask. I do not think it has the same structure in taking a medical history, because you can jump and go back. It does not really look like reality. […] Advantages with the computer cases were that you also got to see information from physical examination and lab results.”
**VP application**	
Learning facilitators	
Student #21 (female)	“Almost every time I have met a [real] patient, it has been by myself or with a physician. […] you cannot discuss [with a real patient] the same way as with a robot or a computer because of ethical aspects […]. I think that a conversation with a computer or a robot is good for [training] collaboration and discussion with other colleagues.”
Student #23 (female)	“It was good that there were only two or three [students] who sat together, so you could discuss, just like you do in real life.”
Student #18 (male)	“[…] there are three people talking to one patient. […] There was a little discussion among us, which we can’t have in real life.”
Student #17 (male)	“[…] it was sometimes difficult to get answers [from the robot] on exactly what you wanted, and then it was difficult to move on or go back.”
Student #21 (female)	“The first case [on the computer-based platform] was a bit difficult to understand, so you just want to click on all the questions. But the more you did, the more you got into it.”
Student #2 (male)	“A thought that hits me is that you can run them [the computer-based cases] before. […] and then when you feel a little more secure, you could run the robotic patients. Then you would successively raise the degree of difficulty.”
Student #20 (male)	“It was sometimes difficult to formulate the questions in English. […] We have learned how to take a medical history and how to describe different symptoms in Swedish.”
Skill development	
Student #21 (female)	“I started to ask ‘tell me why you’re here or how you’re feeling today’. […] sometimes more information came up that made me change my mindset from what I had in mind in the beginning. […] It was also important that, especially with the robot case, to remember what the patient had said. […] I practiced taking notes while the patient was talking.
Student #11 (male)	“[…] you got to think what type of information you need [from the robot], and really dive in in order to get the medical history.”
Student #10 (male)	“With the robot, there were no pre-determined questions. […] There is a difference between being able to choose between a list and thinking ‘this is a reasonable question’ and keeping them in your head. […] The robot really made me remember everything.”
**Strengths and weaknesses**	
Technical limitations	
Student #6 (male)	“It wasn’t possible to find out about laboratory tests, heart and lung examinations, which means that it [the robot] was limited in terms of information.”
Student #8 (male)	“It is something with meeting a patient or a robot, which I think attaches more, but it is a subjective feeling. […] It feels like you get more out of the meeting [with the robot] instead of clicking on a specific question.”
Student #20 (male)	“When you talked to the robot you had to say everything in a coherent sentence, […] sometimes, if you paused and thought in the middle, it interrupted you and answered the question. That felt like a barrier.”
VP potential	
Student #14 (male)	“I think it [the robot] is a very good complement to clinical rotations. […] a lot of it is to get started and conduct a consultation with a patient as a student. You could train on a virtual patient, so that you have some kind of model that works for you when you get to see real patients later. Also, you can decide exactly which diagnosis you want [to practice on]. […] With these virtual patients, you can standardise the diagnoses you get to train on.”
Student #11 (male)	“Another advantage is that if you are not sure how to treat a patient and if you don’t really know how […] to decide which questions to ask or which solutions apply, the virtual patient cases become a safer alternative. If you do something wrong, there’s no risk of hurting someone.”
Student #4 (female)	“If you think about how clinical rotations are in reality […], you often end up doing administrative things most of the time rather than meeting patients. So, in that way, if you would have access to virtual patients or robots, then you would get more used to meeting patients and you could do that more independently than if you only meet a regular patient. With a real patient you always have to go to your supervisor and ask ‘what should I do now?’ […]. It takes time from the supervisor, and it might not be as effective as when you deal with the robots.”

Realism was another essential component of authenticity. Students perceived the social robotic platform as providing a more realistic experience compared to the computer-based platform. The social robotic platform enhanced the proximity to real scenarios, the authenticity of interactions, and the feeling of enacting as the attending physician. Several students felt that the robotic cases allowed them to train on what they would practice in future real-world clinical settings (Table 2).

The sub-theme of medical history and examination highlighted the differences between the two VP platforms in terms of training in obtaining medical information. The social robotic platform was perceived as better for training on prompting relevant responses from the VP, whereas the computer-based platform allowed for simulating physical examinations, laboratory testing, and other parts of the clinical investigation. While the robot case provided more opportunities for communication and specific questioning, the immediate access to medical data in the computer-based platform was perceived as beneficial by some students (Table 2).

Collectively, the theme “authenticity” in VP interactions emerged as a multifaceted concept, with communication, realism, and medical history and examination all contributing to the students’ perception of an authentic learning experience. The social robotic platform was overall considered superior in providing an immersive and realistic experience, while a strength with the computer-based platform was the provision of structured access to medical data.

### VP application

The theme “VP application” was divided into two sub-themes, i.e., (i) learning facilitators and (ii) skill development.

Learning facilitators were identified as key components that enhanced the learning experience and acquisition of CR skills with VPs. Students reported that participating in a small group of 2–3 students facilitated better learning through discussion and collaboration. The combination of different VP platforms, such as the social robotic platform and the computer-based platform, was also seen as beneficial, with each platform training different skills. The social robotic platform was found to be better for training medical history obtainment, while the computer-based platform was more suitable for identifying key indications for specific medical procedures. The experience from the user’s perspective and the level of complexity were also reported as important factors; the students needed varying long time to get used to the operation of the VP platforms. The two platforms presented varying levels of difficulty, with the social robotic platform requiring users to generate their own questions and the computer-based platform providing pre-written questions. This difference allowed for a progression in learning, with the computer-based platform being more suitable for students with less knowledge within rheumatology and the social robotic platform being more attractive for students with consolidated theoretical background in the field. Language was another factor that influenced the learning experience, as some students mentioned that the VPs being in English, their second language, could have impeded acquirement of certain skills, as they had learned how to obtain medical history and describe symptoms in their mother tongue, i.e., Swedish. Representative quotes are illustrated in Table 2.

Skill development was the second sub-theme identified within “VP application”. Students reported that the VP platforms allowed them to develop and train various skills, with the social robotic platform being particularly advantageous for CR training. The robotic VPs encouraged students to build up reasoning based on symptoms and anamnesis, formulate spontaneous questions, and adapt their thinking based on new information provided by the patient. The lack of backward tracking of the interaction in the robotic platform also trained students in taking notes and in remembering information provided by the patient (Table 2).

In summary, the theme “VP application” highlighted the importance of learning facilitators and skill development in the context of VP interactions. Small group discussions, the combination of different VP platforms, the user experience, the level of complexity, and language all played a role in shaping the learning experience. The social robotic platform was seen as a valuable tool for developing CR skills and other essential skills for physicians.

### Strengths and limitations

The theme “strengths and limitations” was divided into two sub-themes, i.e., (i) technical limitations and (ii) VP potential.

Technical limitations were identified as factors that hindered the learning experience with VPs. Students reported that the lack of physical examination and laboratory testing options in the social robotic platform hindered some aspects of CR training. In this respect, the cases within the computer-based platform were perceived as more complete. The computer-based platform was also found to have limitations, as it did not allow students to generate their own questions, leading to a less interactive experience. The dialogue within the social robotic platform was sometimes perceived as mechanical. Pauses during formulation of questions to the robot resulted sometimes in interruptions by the robot to generate a response. This led students to pose shorter and less complicated questions compared to what they might have asked in a real patient consultation. Representative quotes are illustrated in Table 2.

Despite these limitations, the sub-theme of VP potential highlighted some opportunities and future applications of VPs in medical education. Students pointed out that VPs can be an efficient tool for being exposed to representative patients and accumulating experience, which often can be difficult to ascertain in real-world settings. The social robotic platform was seen as particularly useful for teaching medical history obtainment during early stages in the medical curriculum, providing a safe environment for students to practice CR without the risk of harming real patients. Students also emphasised that VPs could offer a more standardised learning experience. This in turn could allow them to train on a variety of diagnoses that they might not encounter during clinical rotations. Hence, VPs were seen as a valuable complement to real patient interactions, providing more opportunities for independent learning and demanding fewer supervisor resources (Table 2).

Overall, the theme “strengths and limitations” highlighted the technical limitations and potential applications of the social robotic and the computer-based platforms in medical education. While some limitations of the social robot were identified, such as the lack of physical examination options and, at times, mechanical dialogue owing to response delays and repetitions, the potential of the platform to provide patient encounters that are representative for the area of training, safe learning environments, and standardised training opportunities was also recognised.

## Discussion

By means of in-depth interviews and thematic analysis, this qualitative study aimed to investigate potential added value with an LLM-enhanced social robot compared to a conventional computer-based VP platform in training CR skills among medical students undertaking clinical rotations within rheumatology. The students found the social robot to be more authentic and engaging than the computer-based platform. They considered it superior for training CR skills, particularly those related to patient consultation, as well as aspects of communication. Most students described that the social robotic platform encouraged them to generate questions and form hypotheses. This, in turn, led them to actively consider how to proceed with the consultation. The students’ active engagement in the consultation process was a key factor in perceiving the social robot as authentic.

To our knowledge, this project is the first to investigate VP simulation using a combination of an LLM with social robotics. Although direct comparisons with previous studies are not feasible, our findings are consistent with research emphasising the importance of interactivity and complexity in VP platforms for achieving specific learning outcomes. For instance, Plackett et al. [32] suggest that highly interactive VPs enable educators to better evaluate students’ CR skills through direct observations of their abilities compared with less interactive modalities. Similarly, Huwendiek et al. [33] emphasised the role of VP design features, such as interactivity and feedback, in fostering CR skills. Our study builds upon these findings by showcasing the potential of social robotic platforms in combination with follow-up seminars to enhance the authenticity and interactivity of VP interactions, thus supporting the development of CR skills in medical students.

The students reported that the social robotic platform enhanced their communication skills through features not available in the computer-based platform, such as voice, movement, and the ability to maintain eye contact. They also highlighted the difference between reading pre-written questions and generating questions themselves based on the medical history, noting that the latter more closely resembles a real patient consultation. In fact, this aligns with previous research indicating that a higher level of interactivity and dialogue generation is likely to enhance the achievement of learning outcomes [34, 35].

Students recognised the significant potential of the social robot as an effective complement to their regular clinical rotations, lectures, and seminars, and believed that the platform could be applied to various medical disciplines. They preferred practicing skills with a robot rather than an actual patient, aligning with previous findings emphasising the importance of safe learning environments for practicing clinical decision making [13]. The students’ experiences point to the social robotic platform as a promising tool for the future of medical education as it offers a safe environment for teaching clinical reasoning while being effective, aligned with curricular learning outcomes, and well-received by students.

Educational resources and the availability of quality assurance through dedicated educators and supervisors can vary significantly across countries, care providers, and settings [36, 37]. Utilising VP platforms that closely resemble clinical practice can enhance opportunities to train CR for more students compared with clinical rotations solely, which often rely on the availability of a clinical supervisor. One of the beneficial aspects with the VP platforms that was brought up by students, without any apparent difference between them, was that the educational activity required fewer supervisors to be present. Hence, VP platforms may be beneficial complements in medical education settings with limited supervision resources.

Integrating social robotic VPs into medical curricula could help ensure that all students are exposed to a minimal set of representative cases for a discipline, promoting equity in students’ learning experiences. This is of particular importance in the field of rheumatology [8–10], where some conditions are rare [12]; exposure of medical students to real patients with such conditions can thus seldom be guaranteed during clinical rotations. As highlighted by Cook et al. [38] and Plackett et al. [32], medical students may not always encounter a diverse range of patient cases during their clinical rotations. A social robotic VP can bridge this gap by providing structured, consistent exposure to key clinical scenarios, ensuring that intended learning outcomes are fulfilled. This exposure complements real patient interactions and supports the achievement of curricular goals.

Social robotic VPs offer a more interactive, comprehensive, and complex learning experience compared to other VP platforms. Exposure to highly interactive VP cases may enhance knowledge retention about patients and their conditions, as supported by Edelbring et al. [25], who investigated the non-analytical model of CR and the importance of extensive practical experience for physicians. This addresses the apparent need for training students in identifying and managing complex and heterogeneous rheumatic diseases [8–10], making the discipline of rheumatology a particularly suitable medical arena for training CR through VP simulations.

Beneficial aspects of CR training using VPs can further be explored by delving deeper into the aspects of collaboration and teamwork. Several interviewees mentioned that working in a smaller group consisting of 2–3 students was perceived as beneficial, through increased interaction that would not have occurred if the activity was performed individually. This is in line with previous reports of benefits conferred from collaboration of students in the form of dyads, including increased interaction, better use of the VP platform, and gains regarding CR training [25]. While previous research has shown those benefits of dyads when utilising conventional, computer-based VP cases, similar benefits were also documented in the present study for the social robotic platform. Thus, CR training using VP simulations should be recommended to be conducted in dyads or small groups of students rather than individually. Future studies could further explore collaboration aspects by exposing students from different professions for this novel modality, thus incorporating important interprofessional aspects in CR training, to better prepare students for teamwork around patients across disciplines and professions [39].

The qualitative design of our study has some inherent limitations, such as potential subjectivity in the findings and the risk of bias due to the interviewers not being blinded. It must be acknowledged that this might have disfavoured the reliability and generalisability of our findings. However, we aimed to mitigate these limitations by enrolling a sufficient sample size (*n* = 23) for this qualitative study, which allowed us to generate a rich and diverse dataset and yielded saturation during the thematic analysis. The large volume of data collected increases the likelihood that our findings are representative of the experiences and perspectives of medical students in our setting. Nevertheless, it is important to acknowledge that the results need validation using objective quantitative data, which is warranted in future investigations. Moreover, the findings may not be generalisable to other contexts or populations, and further research is needed to validate them in different settings. Another limitation worth mentioning was the difference in the construct of the platforms; while both may be considered semi-linear, the computer-based platform provided pre-defined questions and answers, whereas the robotic platform required the students to form their own questions which were perceived as prompts by the LLM-empowered robot to generate responses. This may have introduced a bias in our results. However, those differences constituted parts of our questions at issue and were extensively discussed by the interviewees. Lastly, it must be noted that the language of VP cases in this study was English. Some students identified this as a limitation since they had practiced obtainment of medical history in Swedish. The results may therefore not be representative for students with English as their first language or students who do not speak English. However, the use of the English language makes the educational activity available for a wider population of practitioners.

The knowledge that this study contributes about the use of social robotics combined with LLMs as platforms for VP presentation is novel. Further research on this subject is crucial to understanding its implications in a broader context. Future studies could for example evaluate the effectiveness of social robotic platforms in training communication and empathic skills. Additionally, conducting quantitative comparisons between social robotic platforms and conventional computer-based platforms could provide further insights into added value of the social robot in medical education.

In our setting of undergraduate clinical training within rheumatology, we demonstrated that using a social robotic platform enhanced by an LLM offered advantages over traditional computer-based VP platforms in terms of interactivity and authenticity. This is well in line with our hypothesis that the natural movements of the robot would enhance the perception of realism [20], leading to a better learning experience for acquiring crucial CR skills. Despite some limitations, the social robotic platform was perceived as superior to the conventional computer-based platform in terms of CR skill training, authenticity, communication, and the experience of being empathic towards the patient, with a vast majority of interviewees collectively preferring the social robotic platform and recognising its potential for further improvement. We advocate that this technology has merit not only for rheumatology, but also for the undergraduate education within a multitude of other medical disciplines. 

## Electronic supplementary material

Below is the link to the electronic supplementary material.

Supplementary Material 1

Supplementary Material 2

Supplementary Material 3

Supplementary Material 4

Supplementary Material 5

Supplementary Material 6

Supplementary Material 7

Supplementary Material 8

Supplementary Material 9

Supplementary Material 10

Supplementary Material 11

## Data Availability

The data are available from the authors upon reasonable request and with the permission of the Department of Medicine, Solna, Karolinska Institutet (KI).
