# Supplementary material for: Enhancing clinical reasoning skills for medical students: a qualitative comparison of LLM-powered social robotic versus computer-based virtual patients within rheumatology
Source: Rheumatol Int. 2024 Oct 16;44(12):3041–51. doi: 10.1007/s00296-024-05731-0 (PMC11618132; doi:10.1007/s00296-024-05731-0)
Supplement: Supplementary file 11 — Supplementary Material 11 [file 296_2024_5731_MOESM11_ESM.pdf]

Enhancing clinical reasoning skills in medical students: A qualitative comparison of LLM-powered social robotic versus computer-based virtual patients within rheumatology

Alexander Borg, Benjamin Jobs, Carina Georg, Viking Huss, Kristin Waldenlind, Mini Ruiz, Samuel Edelbring, Gabriel Skantze, Ioannis Parodis

SUPPLEMENTARY MATERIAL

TABLE OF CONTENTS

Supplementary Table S1. Consolidated criteria for reporting qualitative research (COREQ): a 32-item checklist for interviews and focus groups. 3
Supplementary Figure S1. Interview guide exploring students' perception of virtual patient platforms regarding acquirement of clinical reasoning skills. 5

Supplementary Table S1. Consolidated criteria for reporting qualitative research (COREQ): a 32-item checklist for interviews and focus groups.

Item	Description
**Domain 1: Research team and reflexivity**	
Personal Characteristics	
1. Interviewer/facilitator	Alexander Borg
2. Credentials	MD; PhD student
3. Occupation	Research intern
4. Gender	Male
5. Experience and training	Experience from PhD courses and supervision from supervisors
Relationship with participants	
6. Relationship established	None
7. Participant knowledge of the interviewer	None
8. Interviewer characteristics	Interest in pedagogical studies and teaching
**Domain 2: Study desing**	
Theoretical framework	
9. Methodological orientation and Theory	Phenomenology
Participant selection	
10. Sampling	Consecutive selection
11. Method of approach	Face-to-face
12. Sample size	23
13. Non-participation	94. Stated reasons were not required.
Setting	

14. Setting of data collection	Clinic
15. Presence of non-participants	None
16. Description of sample	Demographic data, date
Data collection	
17. Interview guide	Used and pilot tested
18. Repeat interviews	None.
19. Audio/visual recording	Audio recording was used
20. Field notes	Field notes were made after the interviews
21. Duration	40–60 minutes
22. Data saturation	Data saturation was discussed after interviews
23. Transcripts returned	Not returned
**Domain 3: analysis and findings**	
Data analysis	
24. Number of data coders	3
25. Description of the coding tree	Provided in the manuscript
26. Derivation of themes	Derived from the data
27. Software	NA
28. Participant checking	None
Reporting	
29. Quotations presented	Participant quotations presented using participant number and gender
30. Data and findings consistent	Data was consistent as presented
31. Clarity of major themes	Major themes presented in tables and manuscript text
32. Clarity of minor themes	Minor themes are presented and described in tables and manuscript text

Supplementary Figure S1. Interview guide exploring students' perception of virtual patient platforms regarding acquirement of clinical reasoning skills.

Introduction

- Presentation.
- The purpose of the interview is to explore perceptions and experiences from the usage of virtual patient cases through an AI-driven social robot compared with a conventional semi-linear computer-based platform, for the training of clinical reasoning skills.
- Information about the amount of interviews and recruitment.
- Contact information, neutrality and pseudonymity: Personal information will be saved on locked servers in a coded format prior to analysis. Analysis-ready datasets will be pseudonymised. Results will be reported at an aggregated level.
- Informed consent form (ICF).

Background

- Could you tell me about your background? (i.e. sex, age, previous educational background if any, country of origin, current university)
- Which semester are you attending at the medical programme at this moment and what clinical rotation or course are you attending right now?

Virtual patient cases

- Where were you located when you performed the cases (if not at the clinic)?
- What were your perceptions and thoughts prior to this activity? Did you prepare for the activity in any way?
- Did you experience that you had sufficient time for performing the educational activity? Would you appreciate shorter/longer time allocated?
- How ”close” did you feel towards the patient? Do you experience that this differed across specific cases and/or the two platforms?
- Did you experience that you had the role of a physician during a patient encounter? Please elaborate on your thoughts regarding the patient encounter?
- Which skills did you experience that you were able to practice the most during the virtual patient activities?
- Quality of the patient encounter: Can virtual patient cases be a complement to meeting real-life patients in the clinic for teaching purposes?
- Could you please elaborate what the word clinical reasoning means to you?
- Is there any other instance, or anything else you can think of, where virtual patient simulations can be of benefit if implemented? For example, a formative test, an examination, learning through failure in a safe environment, etc.

Comparisons between the platforms regarding clinical reasoning and learning experience

- Could you please describe your experience with the conventional semi-linear virtual patient simulation platform? What aspects of the platform stood out to you in terms of facilitating clinical reasoning skill development?
- Could you please describe your experience with the large language model-based social robotic platform for virtual patient simulations? What aspects of the platform stood out to you in terms of facilitating clinical reasoning skill development?
- How would you compare your experience with the two platforms in terms of supporting the acquirement of clinical reasoning skills?
- In your opinion, what are the main strengths and weaknesses with each one of the two platforms with regard to acquirement of clinical reasoning skills?
- How do the potential differences impact the overall learning experience?
- How did you perceive the level of engagement and immersion offered by each platform during virtual patient simulations? Do you believe one platform provides a more realistic and authentic experience for practicing clinical reasoning skills? Please elaborate.
- Can you share any specific instances where either one of the virtual patient platforms positively influenced your ability to develop and apply clinical reasoning skills? How did this differ between the two platforms? Please elaborate.
- Based on instances that you remember, which of the two platforms benefitted your training towards acquirement of clinical reasoning skills the most?
- From your perspective, what role do virtual patient platforms have in enhancing the learning process towards acquirement of clinical reasoning skills compared with real patient encounters? Do the two different virtual patient platforms contribute to this enhancement differently? If yes, in which aspects? Please elaborate.
- Were there any challenges or limitations that you encountered when using either one of the two virtual patient platforms? How did these differ between the two platforms?
- In what ways do you believe the large language model-based virtual patient platform can be improved to better support the development of clinical reasoning skills? Are there any features or functionalities that you would like to see enhanced or added?

- From an instructional standpoint, how do you think educators can leverage the unique aspects of each platform to optimise the learning experience for clinical reasoning skill development?

General comparisons

- Overall, based on your experience with both platforms, which one do you believe holds greater potential to effectively train and improve clinical reasoning skills? Please elaborate.

- Overall, do you believe that virtual patient simulations constitute a good educational tool to practice clinical reasoning skills? Please elaborate.

- Overall, do you believe that one or the other platform is more enjoyable in terms of VP-practice, i.e. acquirement of clinical reasoning, medical history taking etc. Please elaborate. Probing question: Please mention aspects in which one platform was more enjoyable.

Conclusion

- Do you have anything to add or share in relation to your experience with the virtual patient encounters?
- Do you have anything else to add in general?
- Do you have my contact information?
- Thank you for your participation in the project!
